# A Kalman Filter Approach for Estimating Tendon Wave Speed from Skin-Mounted Accelerometers

**DOI:** 10.3390/s22062283

**Published:** 2022-03-16

**Authors:** Dylan G. Schmitz, Darryl G. Thelen, Stephanie G. Cone

**Affiliations:** 1 Department of Mechanical Engineering, University of Wisconsin–Madison, Madison, WI 53706, USA; dgschmitz@wisc.edu (D.G.S.); sgcone@wisc.edu (S.G.C.); 2 Department of Biomedical Engineering, University of Wisconsin–Madison, Madison, WI 53706, USA

**Keywords:** Kalman filter, shear wave tensiometry, tendon mechanics

## Abstract

Shear wave tensiometry is a noninvasive approach for assessing in vivo tendon forces based on the speed of a propagating shear wave. Wave speed is measured by impulsively exciting a shear wave in a tendon and then assessing the wave travel time between skin-mounted accelerometers. Signal distortion with wave travel can cause errors in the estimated wave travel time. In this study, we investigated the use of a Kalman filter to fuse spatial and temporal accelerometer measurements of wave propagation. Spatial measurements consist of estimated wave travel times between accelerometers. Temporal measurements are the change in wave arrival at a fixed accelerometer between successive impulsive taps. The Kalman filter substantially improved the accuracy of estimated wave speeds when applied to simulated tensiometer data. The variability of estimated wave speed was reduced by ~55% in the presence of random sensor noise. It was found that increasing the number of accelerometers from two to three further reduced wave speed errors by 45%. The use of redundant accelerometers (>2) also improved the robustness of wave speed measures in the presence of uncertainty in accelerometer location. We conclude that the use of a Kalman filter and redundant accelerometers can enhance the fidelity of using shear wave tensiometers to track tendon wave speed and loading during movement.

## 1. Introduction

Shear wave tensiometry is an emerging biomechanics tool for measuring in vivo tendon forces during human movement [1]. Traditionally, muscle-tendon forces are indirectly estimated from ground reactions and body segment kinematics. This inverse approach requires specialized equipment and modeling assumptions, making it ill-suited to evaluate muscle-tendon loading outside the laboratory. In contrast, shear wave tensiometry assesses tendon shear wave speed with skin-mounted excitation and accelerometers. Muscle-tendon forces can be inferred from shear wave speed, as predicted by a tensioned beam model [1]. Prior studies have used shear wave tensiometry to characterize Achilles and patellar tendon forces during walking both in and outside of the laboratory [2,3,4].

Shear wave tensiometry depends on the ability to accurately sense and characterize wave travel time between accelerometers. Tensiometry uses a mechanical tapper to impulsively excite a transverse wave and skin mounted accelerometers to sense the wave arrival at fixed points along the tendon. Wave travel time can be estimated by finding the time to peak wave arrival at fixed spatial locations [5]. Alternatively, one can find the time shift that maximizes the normalized cross-correlation of two accelerometer signals [1], as implemented in prior studies [2,3,4]. A challenge with this approach is the distortion of the shear wave, which can occur due to dispersion, damping, and measurement noise [6,7]. Distortion results in decorrelation of the wave signals as observed at two spatial locations and thereby introduces uncertainty into the estimated travel time [8,9]. Decorrelation is most concerning at high tendon loads, when in vivo wave speeds can be as high as ~100 m/s [1].

Spatial and temporal averaging approaches can be used to enhance wave tracking in biological tissues. For example, shear wave elastography, which uses high frame rate ultrasound to track waves, employs an array of transducer elements to sense wave arrival at many fixed spatial locations [7,10,11]. Least squares approaches are employed to estimate a wave speed that best corresponds with wave arrival measures [12,13]. When wave speed is varying, it is also feasible to measure the temporal shift in wave arrival between successive taps. An advantage of comparing successive wave trajectories at a fixed sensor is that the signals are highly correlated, as dispersion and spatial filtering effects at a single location are mitigated relative to measurements between sensors. Lack of decorrelation should therefore improve the accuracy with which the change in wave arrival time can be estimated.

A Kalman filter can potentially be used to fuse redundant spatial and temporal accelerometer measurements of wave propagation in a tendon. A single tap provides an estimate of wave travel time between pairs of sensors, albeit noisy due in part to signal decorrelation. Successive taps can be used to estimate the change in travel time to a single accelerometer, although that is not sufficient information to calculate the absolute arrival time. A Kalman filter [14,15] can be used to couple these measures with a model of the system and noise processes at play. The change in wave arrival is used to estimate absolute wave arrival times, which are then updated based on statistical comparisons to noisy measures of wave travel. The output of the filter is an optimized estimate of the wave travel time, and hence the speed of the traveling shear wave. In the case of shear wave tensiometry, Kalman filtering may prove beneficial for mitigating fluctuations in the computed shear wave speed due to noise and wave distortion.

The objective of this study was to implement and test a Kalman filtering approach for processing shear wave tensiometry measurements. We compared the accuracy of estimated wave speeds to those obtained using a previously established cross-correlation technique [1]. In the following sections, we describe the Kalman and cross-correlation approaches to estimating wave speed and then compare the sensitivity of each to sensor noise and sensor spacing error. We then demonstrate the application of the Kalman filtering approach for estimating the Achilles tendon wave speed during human walking and running.

## 2. Materials and Methods

### 2.1. Shear Wave Tensiometer

**Figure 1:**
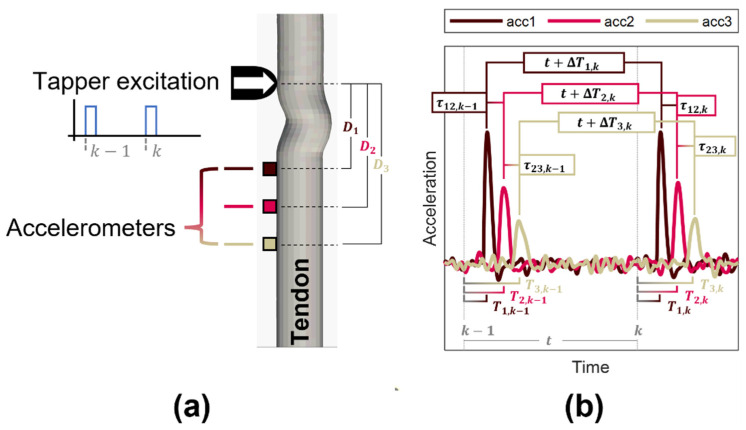
(**a**) A shear wave tensiometer fundamentally consists of an impulsive excitation which induces a shear wave in a tendon or other tissue. The wave travels longitudinally along the tissue. The wave is detected by an array of accelerometers that are adhered to the skin over a tendon. (**b**) An example of the wave measured at three accelerometers. Relevant times are defined for each accelerometer and pair: the variable T is the arrival time of the wave at each accelerometer, τ denotes the wave travel time between consecutive accelerometers, and ΔT indicates the change in wave arrival time between consecutive excitations at a single accelerometer.

Shear wave tensiometers consist of a mechanical tapper and miniature accelerometers in series. Tensiometers are secured over a superficial tendon and used to induce and track shear waves in the tissue (Figure 1). Waves are induced via a mechanical tapper that impulsively taps at a fixed rate (e.g., 100 Hz). Wave propagation is detected via skin-mounted accelerometers that are maintained at fixed relative distances via a silicone array. Prior studies have used two accelerometers to measure the wave travel time between accelerometers, and hence the wave speed. In this study, we considered the use of an arbitrary number (n≥2) of accelerometers sampled at 50 kHz. For simplicity, our figures illustrate the case for n=3 accelerometers, but the general approach holds for an arbitrary number of accelerometers.

### 2.2. Wave Travel Times

For each tap, wave travel times between successive accelerometers are computed using a cross-correlation approach [1,2,3,4]. A template is first defined as the more proximal (to the tapper) accelerometer signal (i) within a window (typically 1–3 ms) after the tap onset. A search region is defined as the more distal accelerometer signal (j) within a window extending from the tap onset to a time that is inclusive of the maximal potential wave travel time. The normalized cross-correlation, r(τ), is then computed between the template and search windows for all candidate discrete travel times τ. The discrete travel time that produces the peak correlation is determined. A cosine fit to the cross-correlations adjacent to the peak is used to obtain a sub-sample estimate of the wave travel time (τ) between accelerometers i and j for tap k, referred to as $\tau_{ij,k}$ [16].

A similar cross-correlation approach is used to compute the change in wave arrival at a single accelerometer (i) between successive taps k−1 and k. The template is defined as the accelerometer signal within a window (1–3 ms) after tap k−1 onset. The search region is defined as the same accelerometer signal within a window that starts before tap k onset and is inclusive of the minimum and maximum potential changes in wave arrival time. The normalized cross-correlation, r(ΔT), is computed between the template and search windows for all candidate discrete changes in wave arrival times ΔT. After finding the shift of peak correlation, a cosine fit is used to obtain a sub-sample estimate of the change in wave arrival for accelerometer i, referred to as $\Delta T_{i,k-1}$.

### 2.3. Kalman Filter

**Figure 2:**
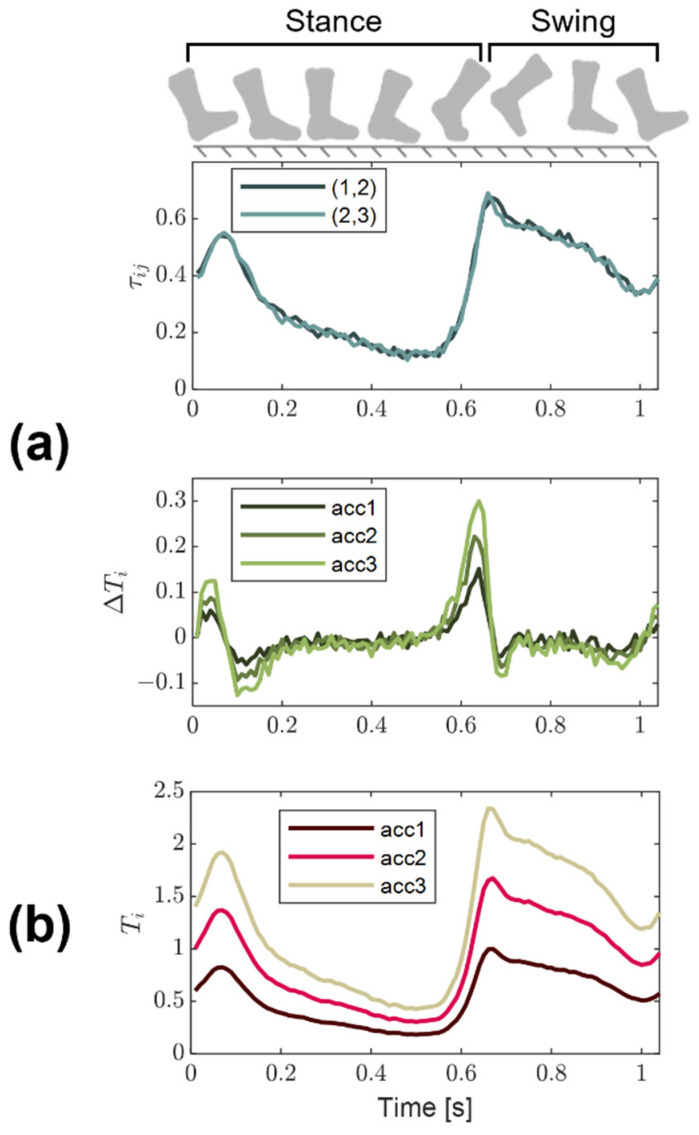
(**a**) An example of the Kalman filter measurements, inputs, and outputs for a tensiometer with 3 accelerometers. The wave travel time ($\tau_{ij}$) between successive pairs of equally spaced accelerometers was generally similar, with slight differences due to noise in the system. The change in wave arrival ($\Delta T_{i}$) at an accelerometer between tap events was centered around zero, with larger changes in arrival for accelerometers further from the tapper. (**b**) The estimated arrival time of the wave at each accelerometer ($T_{i}$) as computed by the Kalman filter.

We implemented a Kalman filter approach to fuse the measures of wave travel time between accelerometers and change in wave arrival at a given accelerometer (Figure 2). The state variables are the vectors of shear wave arrival times (T) at each accelerometer. The control input is a vector of the change in arrival times (ΔT) of sequential waves for each accelerometer. The arrival of the shear wave at an accelerometer i at time k is then deconstructed into the wave arrival at time k−1 plus the change in wave arrival at time k. A state extrapolation equation for an accelerometer i was thus described by the following representation:

(1) $$T_{i,k}=T_{i,k-1}+\Delta T_{i,k-1}+w_{k},$$

with $w_{k}$ representing the process noise. Given n accelerometers, a state transition matrix (F) and the control matrix (B) were defined as n×n identity matrices. This results in the following Kalman filter model for the system:

(2) $$T_{k}=FT_{k-1}+B\Delta T_{k-1}+W_{k},$$

where $W_{k}$ is the process noise vector.

The observed states for the system are the estimated wave travel time ($\tau_{ij}$) between successive pairs of accelerometers. The wave travel time represents the difference in wave arrival time at accelerometers i and j. Thus, an observation equation is given as:

(3) $$\tau_{ij,k}=-T_{i,k}+T_{j,k}+v_{k},$$

where $v_{k}$ is the observation noise pertaining to τ. An additional constraint can be included assuming the wave speed is constant along the tensiometer. Given the distance *D* of each accelerometer from the tapper is known, the wave speed equation results in the following constraint:

(4) $$0=D_{j}T_{i,k}-D_{i}T_{j,k}+v_{k},$$

where $v_{k}$ is the observation noise pertaining to noise in the wave arrival and uncertainty in the accelerometer position. Collecting the coefficients into the observation model matrix H allowed us to represent the complete set of observation equations as:

(5) $$z_{k}=HT_{k}+V_{k},$$

with $z_{k}$ representing the vector of observations and $V_{k}$ contains the observation noise vector.

The process noise $W_{k}$ and the measurement noise $V_{k}$ are based on the diagonal, n×n noise covariances $Q_{k}$ and $R_{k}$, respectively. To numerically implement the equations, we estimated the noise covariances with numerical simulations as described in Section 2.5.

**Figure 3:**
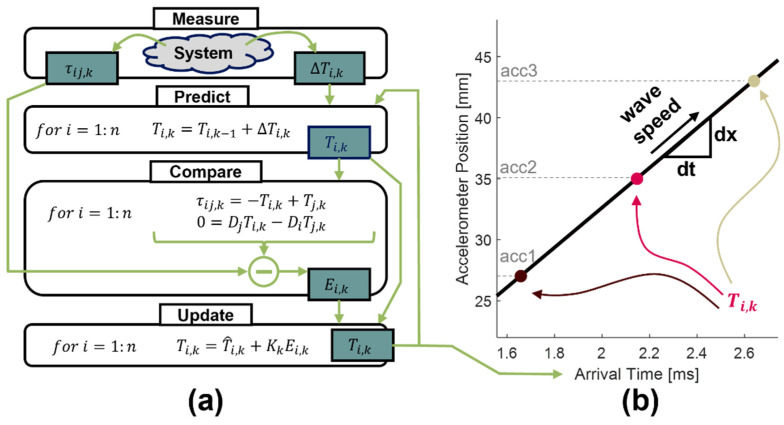
(**a**) Flowchart depicting the iterative Kalman algorithm used to compute wave arrival times at each accelerometer location. (**b**) A weighted least squares regression was used to determine the best wave speed based on the Kalman arrival times. The weights of each arrival time were based on a secondary output of the Kalman filter—the estimated state uncertainty.

The output of the Kalman filter (Figure 3a) is a vector of wave arrival times of length n, one for each accelerometer. The wave speed can then be estimated as the slope of a least squares regression of accelerometer position versus wave arrival time, weighted by the estimated state uncertainty at each time point (Figure 3b).

### 2.4. Shear Wave Tensiometry Simulations

We created a numerical simulation of tensiometry data to investigate the potential of Kalman filtering for improving tendon wave speed measures. The tensiometry simulation model enabled us to prescribe a known wave speed, simulate accelerometer data in the presence of measurement noise, and analyze the synthetic accelerometer signals using both a cross-correlation and Kalman filter approach. All estimates of wave arrival times and wave speed were then directly compared to those defined in the simulation.

**Figure 4:**
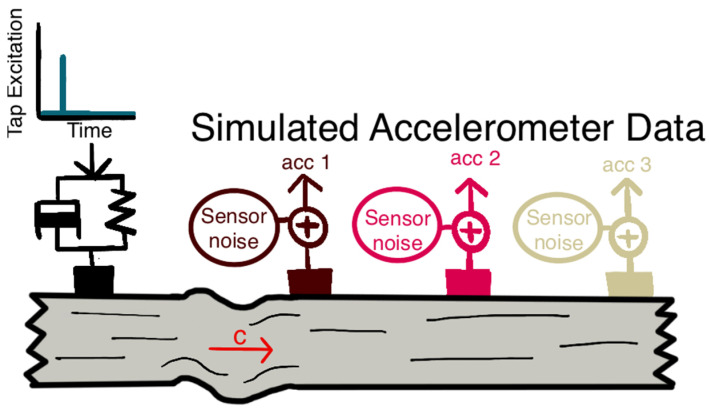
Depiction of the model used to numerically simulate tensiometer data. The tapper-induced wave was represented by the impulse response of an underdamped mass-spring-damper system. The propagating shear wave (traveling with wave speed c) was detected by skin-mounted accelerometers. Sensor noise, represented by band-pass filtered white noise process, was introduced to generate the simulated accelerometer data.

In the simulations, we assumed the motion at an accelerometer could be described as an underdamped second-order impulse response (Figure 4). For a given tap, wave speed and accelerometer position were used to compute the time of arrival of the impulse at each accelerometer. Each accelerometer signal was then simulated as the system impulse response plus sensor noise, which was represented by band-pass filtered, zero-mean Gaussian white noise.

### 2.5. Noise Covariance

**Figure 5:**
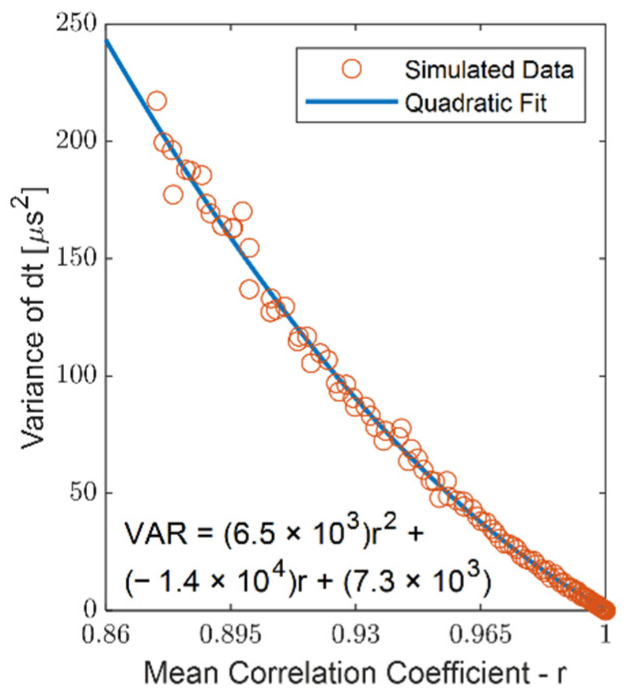
The variance of dt (ΔT or τ in application) with respect to the mean correlation coefficient r is well-approximated with a quadratic fit.

The Kalman filter requires estimates of the measurement and process noise variance for the system. Noise can arise from uncertainty in the wave travel time ($\tau_{ij}$) and change in wave arrival ($\Delta T_{i}$) measures, both of which are obtained via cross-correlation of accelerometer signals (see Section 2.2). We conducted numerical experiments to relate the variance of the measurement (i.e., wave travel time τ) and the control input (i.e., change in wave arrival time ΔT) to the strength of the correlation, as assessed by the correlation coefficient *r*. Cross-correlations were performed on 101,000 simulated tap events, 1000 for each of 101 sensor signal-to-noise ratios (SNR) ranging from no noise (infinite SNR) to 1. For each SNR, the variance in the estimated time shift ΔT and the average correlation coefficient was determined. The relationship between the variance of ΔT and the mean correlation coefficient was well described by a quadratic equation (Figure 5). When processing tensiometry data, we then used this relationship to estimate the variance of ΔT and τ from the correlation coefficient *r*. The variance measures were then used in the Kalman filter to populate the process noise covariance $Q_{k}$ and the measurement noise covariance $R_{k}$. For the constraint equations (Equation (4)), the measurement noise was associated with uncertainty in the accelerometer position. This effect was probed separately (see Section 3.2).

### 2.6. Tensiometry Simulations during Gait

We conducted numerical experiments to probe the capacity of a Kalman filter to improve measures of wave speed. In these simulations, a synthetic shear wave was generated based on the impulse response of an underdamped mass-spring-damper. The undamped natural frequency and damping ratio were randomly selected from a normal distribution with nominal frequencies and damping creating responses representative of measured data (Table 1). The locations of each accelerometer were positioned at fixed locations relative to the wave excitation. Accelerometer signals were contaminated by sensor noise, which was represented by band-pass filtered (100–5000 Hz) white noise processes.

The accelerometer data were simulated for 20 sets of 20 walking strides (total of 400). The nominal accelerometer amplitude and wave speeds for the Achilles tendon over a walking stride were taken from the literature [4]. The sensor signal-to-noise ratio was specified for each set, with the noise standard deviation ranging from 0 (infinite SNR) to half the accelerometer signal amplitude (SNR = 2). The collection of 400 simulations was repeated for arrays of 2, 3, and 4 accelerometers. Simulated accelerometer signals were used to estimate the wave travel time between accelerometers and the change in wave arrival at given accelerometers. These data were subsequently used to estimate the wave speed using the traditional cross-correlation approach [1], and then using the Kalman filter to fuse measures from an array of accelerometers. Errors were evaluated by comparing estimated wave speeds with the wave speed used to generate the synthetic data. Accuracy was characterized by the mean coefficient of variation, which represents the standard deviation in the wave speed estimate normalized to the prescribed wave speed. The coefficient of variation was computed for each time point in the gait cycle and then averaged over the gait cycle.

The effect of an imprecise accelerometer position was explored by simulating tensiometry data over 100 walking strides. For each stride, the true accelerometer position was randomly altered by an amount chosen from a normal distribution (mean = 0 mm, SD = 0.5 mm). The computations of wave speed via cross-correlation and Kalman filtering were performed assuming the nominal accelerometer positions. We repeated these analyses using accelerometer signals from two, three, and four accelerometer arrays. The mean percent error for each stride was calculated by subtracting the prescribed wave speed from the computed wave speed and normalizing it by the prescribed wave speed and then averaging over the stride.

### 2.7. Experimental Protocol

**Figure 6:**
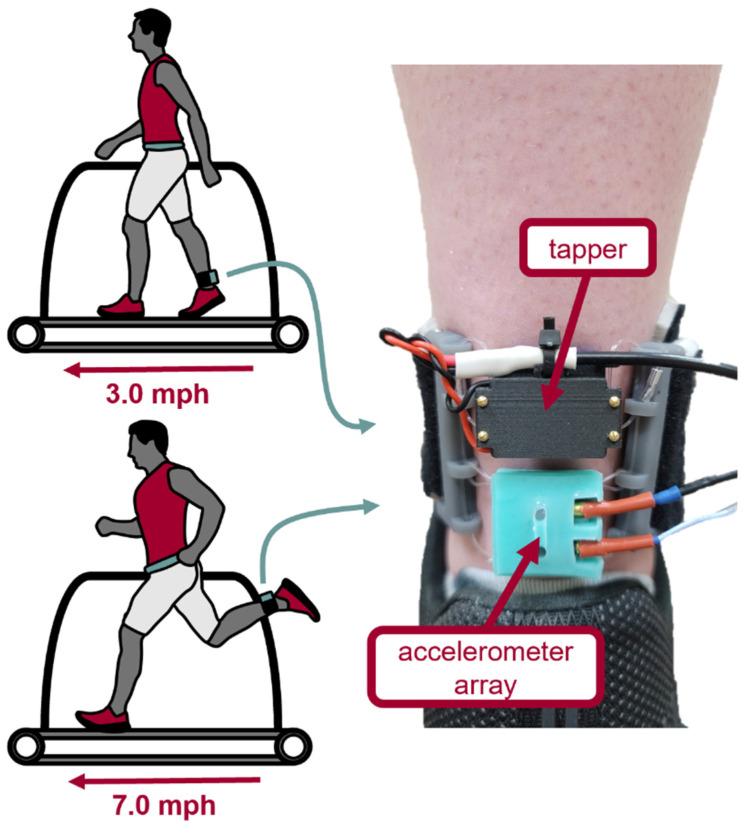
The participant walked (3.0 mph) and ran (7.0 mph) on a treadmill. The shear wave tensiometer was placed over the right Achilles tendon. The tensiometer consisted of a tapper driven by an electrodynamic surface transducer (SparkFun Electronics, Niwot, CO, USA) and two miniature accelerometers (PCB Piezotronics, Depew, NY, USA) affixed in a silicone holder.

Human subject testing was performed with the approval of the University of Wisconsin Institutional Review Board (2018-0487-CP019). Experimental data were collected from 30 s trials with a shear wave tensiometer on the right Achilles tendon of a healthy human subject (n = 1, male, 25 years of age) during walking at 3 mph and running at 7 mph on a treadmill (Figure 6) following previously described methods [1]. Briefly, a shear wave tensiometer consisting of a mechanical tapper and an array of 2 uniaxial accelerometers (spaced 9 and 17 mm from the tapper) was secured over the Achilles tendon. The subject walked and ran while 30 s trials were collected (100 Hz tapping, 0.3 ms duty cycle). All the data were processed with both the cross-correlation and Kalman filter approaches described above.

## 3. Results

### 3.1. Sensor Noise

**Figure 7:**
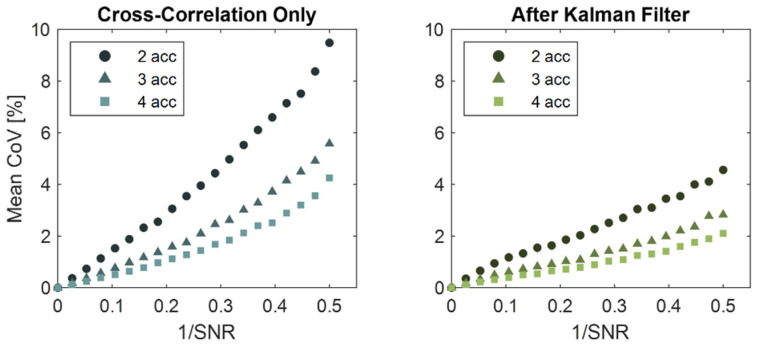
Both the Kalman filter and the use of additional accelerometers reduced errors in wave speed estimates for signal-to-noise ratios (SNR) tested. Plotted is the mean coefficient of variation (CoV—standard deviation of error normalized to prescribed wave speed) for simulated tensiometer data over a walking gait cycle. SNR represents the signal to noise ratio of the simulated accelerometer signals.

**Figure 8:**
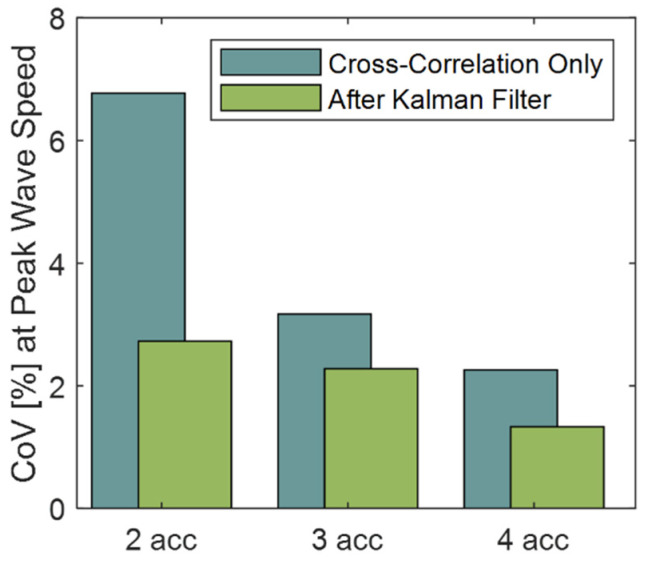
The coefficient of variation (CoV) at peak wave speed was reduced dramatically by using a Kalman filter to process simulated tensiometry data with two accelerometers. Increasing the number of accelerometers further reduced the errors in wave speed estimates. The data shown were from simulated gait cycles with a signal to noise ratio (SNR) of 4.

Simulations with random noise showed that both the Kalman filter and an increased number of accelerometers reduced errors in wave speed estimates (Figure 7 and Figure 8). For all accelerometer sets, the Kalman filter reduced the mean coefficient of variation by up to 55%, with a greater effect at larger noise amplitudes. Similarly, adding accelerometers to the array decreased the effect of random noise for both the cross-correlation only and Kalman filter cases. The mean coefficient of variation was reduced by 47% and 62% for arrays of three and four accelerometers compared to an array of two accelerometers for the cross-correlation only method. Likewise, the average reductions in wave speed errors were 44% and 59% when using the Kalman filter.

### 3.2. Sensor Position

**Figure 9:**
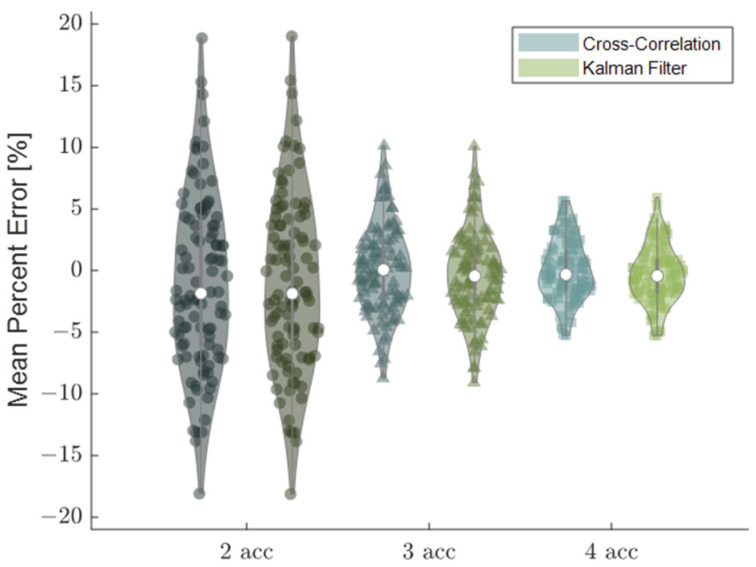
The Kalman filter had no effect on the mean percent error when accelerometer position was randomly varied. However, increasing the number of accelerometers in the array did substantially reduce the wave speed estimate errors.

Varying the sensor positions relative to that assumed within the Kalman filter produced errors in the estimated wave speeds. The mean percent wave speed error, averaged across a simulated walking stride, reached nearly 20%, with the standard deviation of sensor placement set to 5% of the nominal inter-sensor distance (10 mm). The Kalman filter did not reduce these errors, which arise from the biased offset of sensor placement (Figure 9). However, increasing the number of accelerometers used to compute the wave speed did substantially reduce the wave speed error from 20% to 9% (3 accelerometers) and 6% (4 accelerometers), improvements of 55% and 70%, respectively (Figure 9).

### 3.3. In Vivo Tensiometry

**Figure 10:**
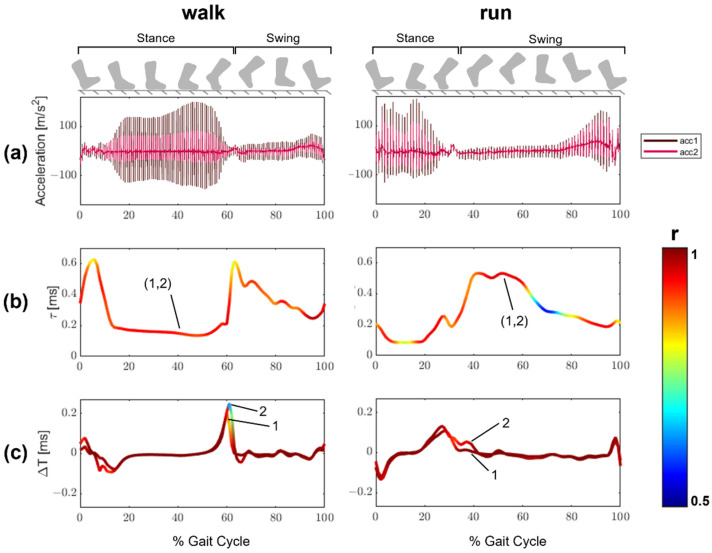
(**a**) Raw tensiometer data (two accelerometers) from the Achilles tendon for an example stride of walking and running. The amplitude of the measured accelerations varies in a regular patter with fluctuations of the muscle state and ankle angle throughout the gait cycle. (**b**) Stride-average estimates of the wave travel time (τ) between accelerometers. (**c**) Stride averaged variations in the change in wave arrival (ΔT) for each accelerometer during walking and running. The line colors in (**b**,**c**) indicate the magnitude of the correlation coefficient (*r*) at that point in the gait cycle. Note that the cross-correlation template for event k was defined using a 2 ms window centered at the arrival time T computed for the previous Kalman iteration k−1.

Tensiometer data collected on an Achilles tendon during walking and running was processed using the same techniques as the simulated data. There were systematic variations in the accelerometer magnitude throughout the gait cycle, with the greatest amplitude accelerometer signals observed when the triceps surae are most active in the stance phase (Figure 10). Accelerometer signals were substantially diminished with plantarflexion during early stance, with signals growing with Achilles tensioning in the late swing. The strength of the cross-correlation of adjacent accelerometer signals varied with accelerometer signal magnitude. The cross-correlation coefficients were generally >0.9 during the stance phase of walking but then were substantially reduced (<0.8) during periods of early swing. The correlation of the same signals between successive taps was consistently stronger, with r > 0.95 through much of both the running and walking gait cycles.

**Figure 11:**
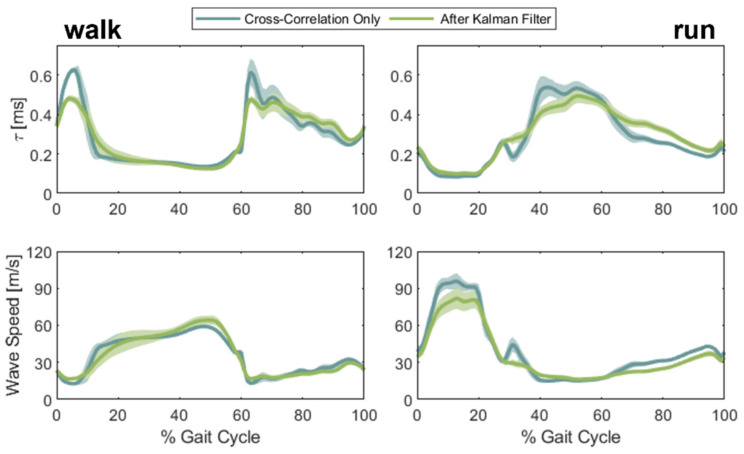
Walking and running data for one subject processed with only the cross-correlation method (blue) and then with the Kalman filter method (green), with one standard deviation above and below highlighted. Application of the Kalman filter resulted in overall lower measured variability both between strides and across the stance and swing phases of gait.

The Kalman filter produced qualitatively similar, but distinct, estimates of Achilles tendon wave speed during both walking and running trials. Since the true value of wave speed was not known for the in vivo data, our analysis of these data is limited to observations of the differences in patterns (Figure 11). During walking, the Kalman filter estimate of wave speed was reduced during early stance (15–25% gait cycle) but enhanced and more distinct during push-off (~50% gait cycle). Further, wave speed fluctuations during the swing phase of walking (65–100% gait cycle) were diminished and smoother. During running, the peak wave speed magnitude with push-off was reduced from 96 m/s to 81 m/s. As seen in walking, the wave speeds during the swing phase of running exhibit less fluctuation and are noticeably smoother when using the Kalman filter. Both the Kalman filter and the cross-correlation technique produced similar estimates of variance across repeat strides, suggesting that either technique can capture the variability inherent in human gait.

## 4. Discussion

This study demonstrated that a Kalman filtering approach was effective at enhancing the accuracy of wave speed measures obtained via shear wave tensiometry. Our approach included two pertinent advances over prior studies [1,2,3,4], which used a pair of sensors to estimate wave speed between measurement locations. First, we used successive excitation events to evaluate the change in wave arrival at a fixed sensor. These metrics were highly reliable as evidenced by high correlations (Figure 10), and hence could be incorporated into a system model to improve estimates of absolute wave arrival time. Second, we showed that the use of redundant sensors, i.e., more than two accelerometers, could mitigate the adverse effects of sensor noise and variable placement on wave travel time estimates. Together, these two advances were shown to reduce variability in estimates of tendon wave speed in numerical simulations. Our numerical results suggested that by doubling the number of sensors and applying a Kalman filter, the mean coefficient of variation of tendon wave speed could be reduced by ~80%, an improvement from 9.6% to 2.1%, compared to cross-correlating signals from a pair of accelerometers.

A predominant challenge in measuring wave speed in tissues is the signal decorrelation that arises with wave travel. Decorrelation can emerge in part as a result of damping, which diminishes the signal amplitude or signal-to-noise ratio. We used numerical experiments to estimate the uncertainty that occurs as a result of decorrelation. For our studies, band-pass filtered noise was added to simulated transient wave signals, and cross-correlation techniques were used to estimate wave travel times. Prior studies in shear wave elastography have applied Kalman filters to ultrasound-based measurements to mitigate the effects of signal decorrelation [17,18,19,20]. Similar to the improvement we saw in wave speed measures, Kalman filtering has been shown to enhance signal-to-noise ratios in strain estimations [21]. Our numerical experiments showed that the uncertainty in travel time could be estimated from the strength of correlation (Figure 5), a relationship that was used to describe the noise processes in the Kalman filter model. Further study is needed to investigate non-noise processes more fully, e.g., dispersion, that can contribute to decorrelation from in situ tensiometry measurements [22,23].

Adding redundant accelerometers was shown to reduce wave speed errors arising from sensor noise and uncertainty in sensor position. Even in the simplest implementation, when independent measures of wave speed are averaged from each consecutive sensor pair, it follows that the effect of random noise would be reduced. The weighted linear regression behaved as a weighted average based on the estimated state uncertainty. Similarly, ultrasound elastography uses a weighted least squares approach across redundant transducer element signals to improve the accuracy of strain and wave speed estimates [7]. There are practical aspects to keep in mind when considering the implementation of redundant accelerometers in a tensiometer. First, there needs to be sufficient space to mount multiple sensors over superficial tendons. Larger accelerometer arrays may be deployable on the Achilles tendon. However, for smaller subjects and shorter structures such as the patellar tendon, the size of the tensiometer may become prohibitively large with redundant accelerometers. A second consideration is the signal attenuation that occurs with wave travel. Attenuation will diminish the signal-to-noise ratio for accelerometers located further from the source, i.e., tapper, excitation. Hence while adding sensors is generally desirable, there are limits to doing this in practice. There were also diminishing reductions in errors as the number of sensors was increased (Figure 7, Figure 8 and Figure 9), such that a compromise on the number of accelerometers to use is reasonable to consider.

The Kalman filter did not further improve wave speed estimates in the presence of random fixed variations in sensor position. Our system model employed the assumption that the distance from the tap excitation input to each accelerometer is known and that wave speed is constant (Equation (4)). A constant sensor position offset reflects a bias error that the Kalman filter cannot readily explain. If the sensor position changed from tap event to tap event, this effect could manifest as a nonlinear distortion. As such, an important factor of tensiometer design is providing well-defined constraints on the sensor positions.

Application of the Kalman filter to in vivo tensiometer data generated Achilles tendon wave speed estimates that exhibited some notable features. During the stance phase of walking, the Kalman filtered wave speed exhibited a more gradual increase than that obtained by cross-correlating signals from two sensors. The gradual increase is more consistent with nominal Achilles tendon loading profiles estimated from traditional inverse dynamics [4]. After toe-off, the Kalman filtered wave speed exhibited less fluctuations than seen in the cross-correlation approach during both walking and running. Accelerometer signal amplitudes decayed precipitously after toe-off due to a change in contact as the ankle rapidly plantarflexes. The signal decay contributed to de-correlation and the less reliable wave speed measures using a cross-correlation approach. The Kalman filter effectively utilized the more reliable tap-to-tap delay information to produce smoother wave speed profiles through this period. While it was not possible to assess absolute wave speed errors with in vivo data, these observations enhanced confidence that the Kalman filter could produce more valid estimates of wave speed trajectories.

There are some limitations to consider in our numerical simulations. First, our simulated noise process did not fully capture the complexity of the in vivo case. Actual accelerometer measurements could be confounded by the effects of spatial filtering, dispersion, and guided wave phenomena [6]. Spatial filtering describes the phenomenon where the frequency content of a signal changes as it travels through a medium. For shear wave tensiometry, it is inevitable that soft tissue damping will act as a filter, attenuating high-frequency signals as the wave travels further from the source. Additionally, the effects of dispersion, which have been observed in shear wave elastography [24,25,26], may have a non-negligible impact on the speed at which waves of different frequencies propagate. There is some evidence of wave-guided behavior in the Achilles tendon [6,24], which could extend to sub-structures. For example, the Achilles tendon consists of three sub-tendons that can exhibit unique loading and hence unique wave propagation behavior [27]. Further studies with high frame rate ultrasound are likely needed to obtain the temporal and spatial data needed to investigate these phenomena and better describe them in simulations of tensiometry. The benefit of the Kalman filter is tempered in cases of significant systematic biases, such as large errors in measured spacing and time-dependent positional data. This emphasizes the importance of designing tensiometry sensors to constrain the distance from the tapper to the accelerometers. Additionally, it is essential to recognize that spatial filtering will occur between consecutive measurements of the propagating wave.

As tensiometry continues to develop, the implementation of physical devices and data processing algorithms advance in parallel. Miniaturization of the tensiometer sensor hardware will allow the distance between each measurement point to be smaller, potentially reducing the extent of decorrelation and spatial filtering. With future applications of tensiometry as a clinical tool in mind, restructuring the processing algorithms toward real-time output will be essential. Since the Kalman filter is an iterative algorithm, this work can be seen as a step towards real-time measures of soft tissue forces. It is important to note that the Kalman methods are independent of the specific excitation and sensing hardware. Finally, as an alternative approach, an investigation into the potential of deep learning methodologies to characterize waveforms and predict wave speeds could be quite powerful [28].

Shear wave tensiometry is an exciting technology with many potential applications and directions for future research. Ongoing work has used tensiometry to quantify Achilles tendon forces during overground walking [3] and is being expanded to include other athletic activities for evaluation and training personalization. Another application takes tensiometers into the orthopedic clinic, where measures of soft tissue loading may be used to guide treatments and objectively track outcomes [29,30]. In each case, high fidelity output from the tensiometer is vital, and this work establishing the benefits of a Kalman filter and redundant accelerometers will strengthen the results we are able to achieve with these devices.

## 5. Conclusions

The results of this study showcased methods for enhancing the fidelity of shear wave tensiometry data by implementing a Kalman filter and using redundant accelerometers. Our simulations suggested that the Kalman filter can mitigate the adverse effects of random sensor noise. In addition, adding redundant accelerometers further increased the efficacy of the Kalman filter, while also reducing the detrimental effects of uncertainty in accelerometer spacing. In practice, we recommend using a Kalman filter when processing shear wave tensiometry data and comparing results to standard cross-correlation methods. The Kalman filter approach may be particularly beneficial for more dynamic tasks, e.g., running and jumping, which can involve high rates of loading and impact events that contribute to noisy periods in sensor measurements.

## Figures and Tables

**Table 1:** Parameters used to define the simulated shear wave tensiometer data.

**Amplitude of measurement**	20 m/s^2^
**Sample rate**	50,000 Hz
**Accelerometer locations** **(relative to excitation)**	[15, 25, 35, 45] mm
**Damping ratio ^1^**	0.5 (0.05)
**Natural frequency of oscillation ^1^**	1600 (100) Hz

^1^ Mean (Standard Deviation).

## Data Availability

The data that support the findings of this study are available from the corresponding author upon reasonable request.
